# Principles and mechanisms of non-genetic resistance in cancer

**DOI:** 10.1038/s41416-019-0648-6

**Published:** 2019-12-13

**Authors:** Charles C. Bell, Omer Gilan

**Affiliations:** 1 Cancer Research Division, Peter MacCallum Cancer Centre, Melbourne, VIC Australia; 2 Sir Peter MacCallum Department of Oncology, University of Melbourne, Melbourne, VIC Australia

**Keywords:** Cancer epigenetics, Epigenetics, Cancer therapeutic resistance

## Abstract

As well as undergoing genetic evolution, cancer cells can alter their epigenetic state to adapt and resist treatment. This non-genetic evolution is emerging as a major component of cancer resistance. Only now are we beginning to acquire the necessary data and tools to establish some of the underlying principles and mechanisms that define when, why and how non-genetic resistance occurs. Preliminary studies suggest that it can exist in a number of forms, including drug persistence, unstable non-genetic resistance and, most intriguingly, stable non-genetic resistance. Exactly how they each arise remains unclear; however, epigenetic heterogeneity and plasticity appear to be important variables. In this review, we provide an overview of these different forms of non-genetic resistance, before exploring how epigenetic heterogeneity and plasticity influence their emergence. We highlight the distinction between non-genetic Darwinian selection and Lamarckian induction and discuss how each is capable of generating resistance. Finally, we discuss the potential interaction between genetic and non-genetic adaptation and propose the idea of ‘the path of most resistance’, which outlines the variables that dictate whether cancers adapt through genetic and/or epigenetic means. Through these discussions, we hope to provide a conceptual framework that focuses future studies, whose insights might help prevent or overcome non-genetic resistance.

## Background

Drug resistance remains the greatest challenge to improving outcomes for cancer patients. Although significant strides towards more effective cancer treatments have been made over the past few decades, most current treatments simply delay the inevitable. There is no doubt that genetic mutations are responsible for many cases of therapeutic resistance.^1–3^ However, our focus on cataloguing these mutations and their functional consequences has caused us to largely overlook the fact that non-genetic/epigenetic changes—that is, changes to gene activity states that occur independently of changes to the underlying DNA sequence^4^ — can also play an important role in drug resistance.^5,6^

The power of epigenetics to modulate changes in cell fate should come as no surprise to any biologist. During the process of development, epigenetic changes enable our entire range of cell types to originate from essentially the same genetic sequence.^7,8^ By comparison with these developmental changes, the epigenetic differences required for a cancer cell to acquire drug resistance appear relatively subtle. Although cancer does not arise through a physiological developmental pathway, epigenetic differences are still present within cancer cell populations and can be stably maintained through cell division. In fact, these differences form the basis of the cancer stem cell (CSC) hypothesis, which refers to normal development to explain the existence of heterogeneous subpopulations within a tumour.^9,10^ Even what might initially appear to be phenotypically homogenous populations of cancer cells are actually heterogeneous, with fluctuations between metastable gene expression programmes reported across a range of cancer types.^11–14^ Taken together, the prevalence of non-genetic heterogeneity in cancer and the ability for epigenetic changes to mediate major differences in cell fate implicate non-genetic evolution as a potential driving force for therapeutic resistance. A number of studies support this hypothesis, clearly demonstrating that non-genetic resistance occurs across a range of cancer and treatment contexts.

In this review, we begin by outlining the evidence for pervasive non-genetic resistance in cancer and highlight the different forms of resistance that have been observed. We then define the two key variables epigenetic heterogeneity and epigenetic plasticity, before exploring exactly how they influence the capacity for non-genetic resistance through Darwinian selection and/or Lamarckian induction. Finally, we discuss the potential interaction between genetic and non-genetic adaptation and define the important factors that dictate which pathway cancer cells follow to acquire resistance. Many of these ideas and concepts will also apply to other aspects of cancer biology, such as metastasis and immune evasion, but for simplicity here we focus on the acquisition of drug resistance. Altogether, this review aims to synthesise our current knowledge into a conceptual framework that assists future investigations of non-genetic resistance in cancer.

## Evidence for pervasive non-genetic resistance

The first examples of non-genetic resistance to be described in cancer involved cancer cells entering a state of drug persistence. Drug-tolerant persister (DTP) cells^14–19^ are broadly similar to persister bacterial cells that are observed in response to antibiotic treatment.^20^ Cancer DTPs arise at a low frequency in the tumour cell population and display reduced growth and altered metabolism, which provides increased tolerance to drug pressure. These cells are genetically indistinguishable from the bulk tumour population, their resistance is reversible upon removal of the drug and, invariably, they arise within populations established from single cell clones, providing irrefutable evidence that persistence is mediated by epigenetic mechanisms.^14–19^ Early studies of DTPs were conducted in vitro, but modelling of resistance in patient-derived xenografts and murine cancer models demonstrates that DTPs also arise in vivo.^21–24^ Although these persister cells are not mitotically active and therefore do not directly result in disease relapse, they can act as a drug-resistant reservoir, providing cancer populations with the opportunity to adapt by acquiring genetic mutations and/or epigenetic changes. Persistence may therefore represent an important predecessor to stable drug resistance.^11,15,25^

In addition to persistence, subsequent studies have identified actual outgrowth of cells that acquire resistance without genetic evolution. In some cases, this resistance is unstable, with reversion of the cells to a drug sensitive state upon drug withdrawal.^26–28^ However, in other cases, epigenetic changes are sufficient to drive mitotically active, stable drug resistance.^13,29–38^ Importantly, together these studies show that non-genetic resistance is not confined to a single cancer type or therapy, suggesting that it is likely to be a pervasive feature of cancer resistance.

Correlative evidence also supports the idea that non-genetic resistance is likely to be widespread. A number of sequencing studies have failed to identify clear evidence of genetic evolution in a large proportion of patients (>40% in some cases) who acquire resistance.^30,33^ Furthermore, chemotherapy has been known for decades to rapidly induce the formation of refractory cell populations that are enriched for CSCs and are less likely to respond to future therapies.^39^ There is also evidence that epigenetic changes can occur independently of genetic changes, with temporal analyses suggesting these epigenetic changes can drive relapse.^40^

Together, these findings demonstrate that non-genetic evolution is likely to be an important component of drug resistance in cancer. In the remainder of this review, we will discuss the variables that determine the capacity for non-genetic resistance and provide insight into the potential mechanisms that enable this form of adaptation.

## Epigenetic heterogeneity and epigenetic plasticity

Before we can explore the potential mechanisms that mediate non-genetic resistance, a clear picture of the factors that contribute to adaptive potential is required. The two key variables that dictate the capacity for a given cancer cell population to undergo non-genetic evolution are epigenetic heterogeneity and epigenetic plasticity.

### Epigenetic heterogeneity

Epigenetic heterogeneity refers to the variability in epigenetic state across a cell population. It is influenced by both cell-intrinsic and cell-extrinsic factors and therefore the degree of heterogeneity will vary according to cancer type, mutational profile and tissue microenvironment.^6^ Analogous to genetic heterogeneity, epigenetic heterogeneity can act as a substrate for Darwinian selection, with a greater degree of diversity increasing the chance that certain cells will display a state capable of surviving and/or adapting to the therapeutic pressure.^36,41–44^ For example, in patients with acute myeloid leukaemia (AML) or breast cancer, individuals with higher epigenetic heterogeneity (independent of genetic heterogeneity) display a poorer prognosis and/or shorter time to relapse.^40,41^

### Epigenetic plasticity

Epigenetic plasticity refers to the capacity of a cell to alter its epigenetic state with a degree of heritability in response to internal or external stimuli. Plasticity is therefore critical for the drug-induced cellular reprogramming that can cause non-genetic resistance. Drug-induced cellular reprogramming can be considered a form of Lamarckian adaptation, as the adaptive changes occur as a direct response to an environmental stimulus. Therefore, plasticity is the driving force for Lamarckian adaptation. For the most part, the nature of epigenetic plasticity is largely unknown, but given that chromatin plays a key role in cell type stability, it appears that the chromatin landscape is likely to be important.^45,46^ Different cell types appear to display different degrees of plasticity, which is likely to influence the capacity of different cancer types to acquire non-genetic resistance.

### The interplay between heterogeneity and plasticity

In instances where plasticity contributes to resistance, heterogeneity must also be involved, as not all cells are capable of surviving and adapting to treatment (see the section on mechanisms of epigenetic resistance, below). For example, in melanoma, heterogeneous, stochastic expression of BRAF inhibitor resistance markers enables the initial survival of a subpopulation of cells, which also has the necessary epigenetic plasticity required to undergo transcriptional adaptation and form a stably resistant population.^31^ We also observed similar results in our model of BET inhibitor resistance in AML:^29,30^ although not intrinsically resistant, the drug-naïve leukaemic granulocyte macrophage progenitor population enriched for leukaemia stem cells showed a greater capacity to adapt than the bulk population. Interestingly, in both of these scenarios, and others, less-differentiated subpopulations acquire drug resistance.^17,18,22,23^ These populations might have increased plasticity due to the less-restrictive chromatin landscape associated with a stem cell-like state (reduced heterochromatin and DNA methylation), which could facilitate activation of a broader range of gene expression programmes in response to therapy. As a result, epigenetic heterogeneity in the form of more plastic, less-differentiated subpopulations might be a common source of non-genetic drug resistance.

It is important to note that heterogeneity and plasticity are not completely independent variables. For example, cells that display greater heterogeneity might do so because the epigenetic state of the population is more plastic. In fact, as mentioned above, plasticity itself can also be a form of epigenetic heterogeneity, whereby different subpopulations have different degrees of plasticity. However, it is conceivable that a population could be highly heterogeneous, but not possess a high degree of plasticity. Likewise, the entire population could be highly plastic, without a high degree of baseline epigenetic heterogeneity. Therefore, epigenetic heterogeneity and plasticity must be viewed as interrelated, yet distinct, variables.

## Potential mechanisms of non-genetic resistance

As mentioned above, non-genetic resistance can come in the form of persistence, unstable resistance or stable resistance. Persistence and unstable resistance are transient processes; however, they can provide an adaptive reservoir for the acquisition of stable genetic or non-genetic resistance.^11,40^ For the most part, current evidence suggests that persistence arises primarily through Lamarckian induction, but in theory it could also occur through Darwinian selection for a pre-existing quiescent subpopulation.^47^ Although there is a clear role for chromatin remodelling in persistence, the exact mechanisms remain unclear and require further investigation.^14,18,34^ Importantly, as these adaptive mechanisms are unstable, drug withdrawal will result in re-sensitisation to the original therapy. Therefore, persistence and unstable resistance can potentially be subverted by cycling treatment regimens.^23,26^ Ultimately, stable resistance has the most widespread and important clinical implications and, accordingly, we will focus our discussion primarily on how cells acquire mitotically active, stable, non-genetic resistance.

### The origins of stable non-genetic resistance

**Fig. 1:**
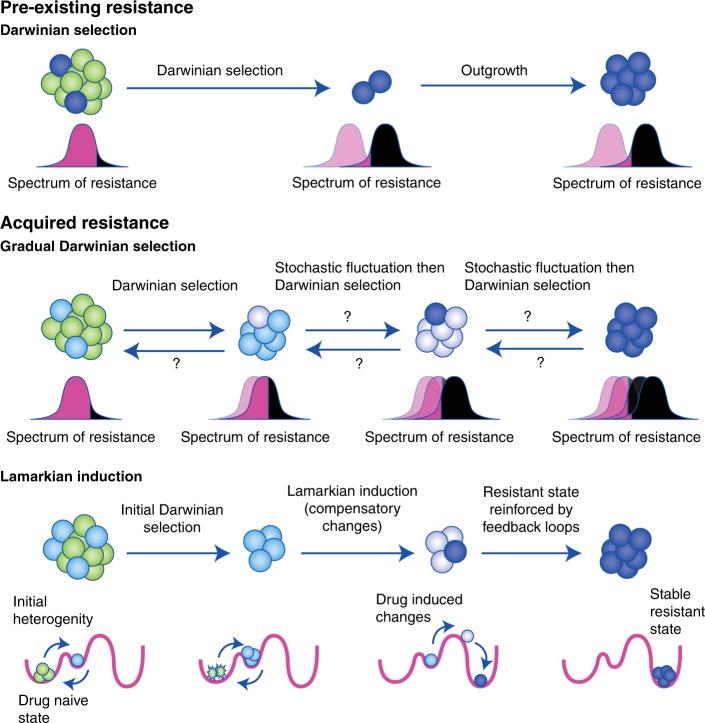
The potential adaptive modes for non-genetic resistance. Resistant states can be either pre-existing or acquired. If there is a pre-existing resistant state, then resistance can emerge through simple Darwinian selection and outgrowth. This mode of adaptation is completely dependent on epigenetic heterogeneity and depends on the pre-existing state being relatively stable. Acquired non-genetic resistance can theoretically arise through either gradual Darwinian selection or Lamarckian induction. Gradual Darwinian selection could occur by selecting for gradually increasingly resistant cells or cells that have an increasingly stable resistance programme. For this mode to apply, the initial cells selected cannot revert spontaneously back to the initial state. In addition, the next generation of cells has to stochastically acquire a more resistant or more stable state. Put differently, the normal distribution of resistance must gradually shift towards resistance with each generation stochastically. Therefore, it is currently unclear how this mode of resistance would be possible. Acquired resistance can also arise through Lamarckian induction. An initial subpopulation in the appropriate epigenetic state is capable of initiating epigenetic changes in response to the drug, which results in the cells moving to a new cell state. We propose that, in general, these drug-induced changes are due to compensation. In some instances, these epigenetic changes could result in the cell transitioning into a new stable cell state, therefore resulting in stable non-genetic resistance.

The origins of stable non-genetic resistance remain elusive, in part due to the currently limited number of instances where this phenomenon has been definitively proven. Theoretically, there are three mechanisms by which stable non-genetic resistance can be achieved. Firstly, the fully resistant state could be pre-existing within a subpopulation of cells, such that the treatment simply selects for this pre-existing stable epigenetic state (Fig. 1). This scenario is a form of non-genetic Darwinian selection and is driven exclusively by epigenetic heterogeneity, with no direct role for plasticity. This adaptive pathway is more likely to be prevalent in cancers/cells that have a high degree of baseline epigenetic heterogeneity, such as pre-existing CSCs.^36,41^ In the absence of pre-existing, fully resistant, stable subpopulations, stable non-genetic resistance must occur through either gradual Darwinian selection or Lamarckian induction (Fig. 1). Although it is relatively easy to characterise pre-existing resistant cells through the use of single-cell technologies, lineage-tracing approaches or elegantly designed clone-based experiments,^32,36,48^ identifying the mechanisms that underpin acquired non-genetic resistance is much more difficult and requires monitoring of the cells during the adaptive transition.

Hypothetically, gradual non-genetic Darwinian adaptation could give rise to drug resistance through incremental selection for outlier cells that display higher drug tolerance (Fig. 1).^10^ Only those outlier cells that express this slightly more resistant programme with a degree of stability/heritability will survive upon subsequent cell divisions. Consequently, this process could theoretically gradually select for the progeny of this original cell that have greater and greater resistance or an increased capacity to maintain expression of the resistance programme. This could in theory eventually produce a population that maintains the programme indefinitely. This idea depends on the assumption that the daughters of this outlier cell will acquire a more stable resistance programme without any influence from the drug, because by definition, Darwinian evolution involves selection on traits that arise independently of the selection pressure. The subsequent progeny of this daughter cell would then have to independently acquire an even more stable resistant state and so on, until the programme is maintained indefinitely (Fig. 1). To us, this process of acquiring resistance seems quite unlikely and inefficient, as the chances that each generation of progeny from the few surviving cells will progressively acquire a more stable resistance programme are low. It is also unclear why the stable resistant state would not be pre-existing in the population, given that there are many more cells present prior to treatment. One possibility is that treatment increases the degree of epigenetic heterogeneity and thereby promotes the stochastic acquisition of resistant states. However, even in this scenario, it is likely that the stable resistant state would be acquired through a single selection event for a fully resistant cell, rather than through gradual selection. Therefore, we argue that gradual Darwinian selection is unlikely to account for most cases of stable non-genetic resistance.

What appears to be a much more likely source of acquired stable non-genetic resistance is Lamarckian induction. By definition, Lamarckian evolution does not occur through selection for pre-existing states; instead, these traits are induced by the environment (which is, in this scenario, the drug).^49^ When considering how Lamarckian adaptation could facilitate non-genetic resistance, it immediately becomes apparent that, for an effective therapy, not all cancer cells survive and adapt to therapeutic pressure. Therefore, some aspect of the initial heterogeneity must provide some of the cells with the capacity to undergo this form of adaptation, while others cannot (i.e. Lamarckian induction occurs together with Darwinian selection for pre-resistant states) (Fig. 1). This leads to two key questions: what part of this initial epigenetic heterogeneity determines whether cells can undergo stable cellular reprogramming? And how does the drug interact with this heterogeneity to trigger stable alterations in cell fate?

New insights that might help to address the first of these questions have emerged by considering what aspect of epigenetic heterogeneity is actually heritable. The Raj laboratory reasoned that, unlike random fluctuations that are not maintained, metastable/heritable changes in gene expression are more likely to enable the eventual acquisition of stable non-genetic resistance.^31,48^ This insight drove them to repurpose the seminal Luria–Delbruck experiment to assess whether the heritability of epigenetic heterogeneity is intrinsically linked to the acquisition of stable resistance. By expanding single cell clones from a melanoma cell line (which they had previously shown acquires stable epigenetic resistance through Lamarckian adaptation) and performing bulk RNA sequencing on each clonal population after a number of cell divisions, they were able to quantify the ‘memory’ of any transcriptional fluctuations. When they correlated these data with the resistance transcriptional programme, they identified a remarkable overlap between genes that undergo heritable fluctuations and those involved in resistance. These findings suggest that non-genetic resistance might be more likely to occur when the population has a pre-existing alternative metastable gene expression programme that it can enter. Interestingly, their analysis demonstrated the existence of only two major alternative metastable states in the specific melanoma population that they studied, whereas studies of melanoma in vivo suggest that there are up to four transcriptional programmes that can facilitate resistance to BRAF or MEK inhibitor combination therapy.^21^ Therefore, each cancer cell population might only have a limited number of possible metastable gene expression programmes that it can exploit to avoid therapy.

Clearly, more widespread analyses of different tumour types and treatments are required to support the link between the presence of pre-existing metastable states and the capacity for stable non-genetic resistance. Although these findings are intriguing and intuitive, it remains possible that any pre-existing heterogeneity (heritable or not) that alters initial drug response^50^ could allow the cell to trigger a cascade of changes that culminates in stable non-genetic resistance, even if a metastable pre-resistant state was not accessible or occupied by the population prior to drug exposure. Therefore, whether metastable/heritable pre-resistant states are a bona fide prerequisite to establish stable non-genetic resistance remains an open question.

### The maintenance/progression to stable non-genetic resistance

So, how does drug treatment interact with this epigenetic heterogeneity to ‘burn in’ the resistant epigenetic state? Put differently, how and why does drug treatment prompt cancer cells to undergo enduring changes in cell fate? To date, studies have simply described the final result of stable non-genetic adaptation, without offering a mechanism that explains why reprogramming actually occurs.^13,29–31^ We propose that the majority of cases of stable non-genetic resistance are mediated by drug-induced activation of compensatory changes. As articulated by Waddington and others many years ago, cells exhibit remarkable robustness, which acts to maintain homoeostasis at steady state and during differentiation.^51^ It is well established that this robustness arises as a result of extensive feedback loops and thresholded biochemical processes.^51,52^ Although cancer cells can have increased heterogeneity and plasticity compared with normal populations,^53^ they do still display robustness. The epigenetic state of a given cancer cell clone is relatively stable and does not drift significantly in a particular direction over short periods of time. Therefore, cancer cells, like normal cells, must possess feedback loops that act to maintain their ‘homoeostatic’ epigenetic state. Upon exposure to an effective drug treatment, a given cancer cell can no longer maintain phenotypic homoeostasis through existing regulatory networks. Consequently, the cells will activate alternative pathways/gene regulatory networks, triggered by feedback loops, in an attempt to circumvent the need for the therapeutic target. Despite these compensatory changes, most of the cells will be killed if the therapy is effective. However, as discussed below, any cells that are in a particular epigenetic state that enables them to withstand the therapy for longer or to activate compensatory programmes more strongly or rapidly might be more likely to survive and be reprogrammed by treatment.

What is the end result of activating these compensatory pathways? If the cancer cells are not sufficiently plastic and/or no alternative stable epigenetic states are accessible, compensatory changes will not trigger the activation of a stable resistance programme. In these cases, if any cells survive, the compensatory changes will be maintained only in the presence of constant drug pressure. This scenario is likely to explain many cases of persistence and unstable non-genetic resistance. Subsequent stochastic fluctuations, further compensatory changes or gradual acquisition of epigenetic memory^54^ might eventually allow this persistent/unstable population to transition to a stable resistant state. Therefore, transient non-genetic resistance might be a common precursor to the eventual formation of stable resistance.^25,38^ However, in other instances, when the cancer cell is sufficiently plastic and alternative stable states are accessible, these compensatory changes could drive the cell directly into another stable cell state (also known as an ‘attractor’ state),^5,55^ resulting in stable non-genetic resistance (Fig. 1).

Under this compensation-based model, the capacity to acquire drug resistance depends upon robust activation of compensatory changes prior to irreversible damage from the drug, making both the dynamics of drug efficacy and dynamics and strength of compensatory signals important factors. As a result, any initial epigenetic heterogeneity that delays the impact of the drug and creates a longer window for alternative states to be activated, or enables more rapid or robust activation of compensatory pathways, could facilitate stable non-genetic resistance.^50,56,57^ For example, cells with a more plastic chromatin environment, such as subpopulations that are less differentiated, will be more permissive to activation of these compensatory pathways and therefore more likely to survive treatment.^36,53,58^ As previously mentioned, pre-existing metastable programmes might also facilitate resistance, as it is more likely that the necessary heterogeneity to withstand treatment will be present if alternative metastable states pre-exist in the population.^31,48^ These pre-existing states might then be reinforced by compensatory transcriptional changes that act to stabilise the previously transient programmes.

To date, there is strong evidence of compensatory mechanisms of resistance in cancers that are dependent on the mitogen-activated protein kinase (MAPK) or oestrogen receptor (ER) pathways. Disruption of these pathways initiates feedback loops that increase their activity and/or activate alternative pathways in an attempt to compensate.^59–62^ We believe that compensatory changes are unlikely to be restricted to therapies that target signalling cascades, which have well-defined feedback loops. For other therapeutic targets, the compensation mechanisms might be more complicated and involve more intermediates, but the intrinsic robustness of regulatory networks will invariably trigger some form of feedback pathway in response to drug treatment. In the future, it will be critical to identify how often these compensatory changes are actually capable of driving a cancer cell into a new, stable epigenetic state, as this will ultimately dictate the prevalence of this form of Lamarckian non-genetic resistance. Likewise, it will be important to determine whether these compensatory changes are mostly generic, such as activation of a stress response pathway,^36,38,39^ or whether they are specific to different types of cancer and therapy.^61^ Improved understanding of these underlying mechanisms could provide us with therapeutic avenues to prevent non-genetic resistance at the source.^21^

## Sources of epigenetic heterogeneity

Since epigenetic heterogeneity is a fundamental property that influences all of the aforementioned mechanisms of non-genetic resistance, it is important to consider its origins. We briefly discuss the sources of functional epigenetic heterogeneity below. For more in depth discussion of the potential causes of heterogeneity, we refer the reader to the following reviews.^6,63^

### Deterministic and stochastic heterogeneity

Briefly, heterogeneity can come in two forms: deterministic and stochastic. Deterministic heterogeneity is a regulated process that provides the cancer with distinct subpopulations—CSCs being a prime example. The sources of deterministic heterogeneity can be either cell intrinsic, such as an in-built functional hierarchy, or cell extrinsic, such as regulation by the tumour microenvironment. Stochastic heterogeneity is variability that arises due to inherent cellular fluctuations or as a result of the imperfection of complex biological systems. Although in some instances stochastic heterogeneity can be exploited for benefit by evolution, it is generally considered an unregulated process.^64^ Sources of stochastic heterogeneity include transcriptional bursting (a process in which transcription from DNA to RNA occurs in ‘bursts’), uneven allocation of cellular contents during cell division, and intracellular fluctuations in other biochemical processes such as metabolism, cytoskeletal changes, enzymatic reactions, etc.^64^

The term stochastic is often misinterpreted to mean that this form of heterogeneity is short-lived (for the duration of a single cell division), yet this is not necessarily the case.^44^ Cellular regulatory networks are organised into highly interconnected modules, within which there is centralised regulatory power.^65,66^ If we envisage a scenario where one of these central well-connected genes is stochastically upregulated, this could lead to co-ordinated changes in other members of this co-regulated module, an observation that has been reported by a number of studies.^31,64,67–70^ Positive-feedback loops could perpetuate these transient changes to provide some heritability to this co-ordinated regulatory network.^71^ In the absence of a deterministic signal, these states are unlikely to be maintained indefinitely and the progeny could eventually revert back to the original state. Such alternate metastable states are likely to be particularly prominent in cancer due to perturbed regulatory networks and disruption of the normal buffering processes. Therefore, stochastic heterogeneity might be the basis for the constantly fluctuating metastable subpopulations observed in a range of cancers, and can act as an important substrate for non-genetic resistance.^31,39^

### Active maintenance of epigenetic heterogeneity?

A fundamental issue is whether cancer cells actively maintain epigenetic heterogeneity to enable them to adapt to changes in their environment^36,47^ or whether they simply exploit the inherent heterogeneity of complex, imperfect cellular systems. In bacterial populations, heterogeneity is actively maintained and exploited through a process called bet hedging, whereby the population assigns a certain proportion of the cells to acquire a different phenotype, which has a lower fitness in the current environment, but which is better adapted if the environment changes.^63,64,72^ The resultant heterogeneity improves the adaptability of the entire population, at the potential expense of the individual cell. It is difficult to imagine how cancer populations could develop and actively maintain such a complex adaptive mechanism through short-term stochastic changes. An alternative explanation is that normal cell types actively maintain heterogeneity, potentially as a regulatory strategy^64^ or to retain adaptability of the entire organism to environmental changes. As cancer cells arise from these normal counterparts, this intrinsic cellular heterogeneity might be co-opted to help resist therapy.

## Interaction between genetic and non-genetic resistance

For simplicity, we have thus far neglected to discuss the fact that genetic and epigenetic mechanisms of resistance are not mutually exclusive, and can actually interact.^47,73^ For example, certain genotypes might have the capacity to undergo non-genetic adaptation, while others do not. In such instances, if we were to perform genomic analysis on the drug-naïve and resistant tumours, we would generally conclude that genetic adaptation was the cause of drug resistance, as certain genotypes are recurrently enriched in resistant tumours. However, for the most part, it has not been assessed whether the genotype that survives therapy also needed to undergo epigenetic changes to become resistant. This has probably contributed to non-genetic evolution being broadly overlooked as a potential resistance mechanism. One way of disentangling genetic and non-genetic contributions to resistance is to test whether the resistance transcriptome/epigenetic state and resistance genotype pre-exist or are acquired through single-cell or clone-based analyses.^30,31^ Such analyses in triple-negative breast cancer supports the idea that a combination of genetic and non-genetic adaptation occurs, at least in some patients.^32^

In our efforts to avoid or overcome drug resistance, it will be critical to obtain a greater understanding of the relative contribution of genetic and non-genetic mechanisms in different cancer and treatment contexts. There is no doubt that different therapies and treatment strategies will be more effective against one of these adaptive pathways than the other.

## ‘The path of most resistance’

**Fig. 2:**
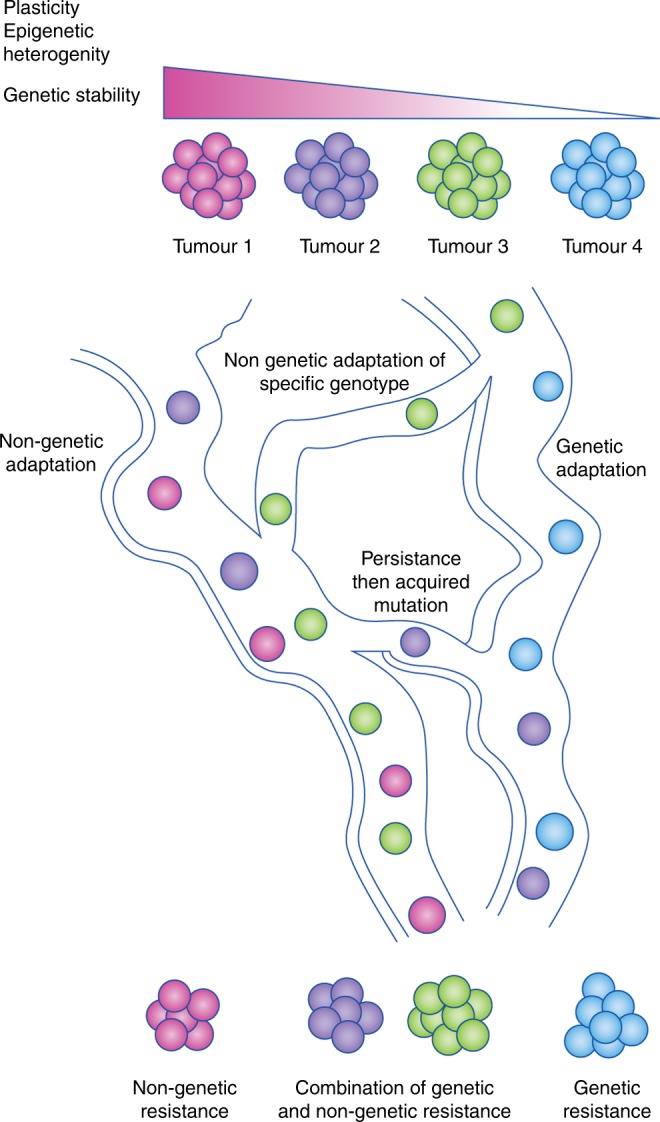
The path of most resistance. Drug resistance can arise through purely non-genetic changes, purely genetic changes, initial non-genetic changes then genetic changes, or non-genetic changes within a particular genotype. Cancer cells will use the adaptive pathway that results in the most rapid or highest degree of drug resistance. The factors that are likely to contribute to which pathway is followed are epigenetic plasticity, epigenetic heterogeneity and genetic stability. Higher plasticity, epigenetic heterogeneity and genetic stability will favour non-genetic adaptation, whereas lower plasticity, epigenetic heterogeneity and genetic stability will favour genetic adaptation. Genotoxic therapies might also promote genetic resistance.

The opportunity for cancer cells to adapt through either genetic and/or non-genetic means raises the question of why different cancers acquire resistance via different adaptive pathways.^73^ We propose that cancer adapts via ‘the path of most resistance’. Logically, whichever adaptive pathway provides the greatest/most rapid selective advantage and, therefore, the easiest way of acquiring drug resistance will be the path that is followed by the population (Fig. 2). A number of variables determine whether the genetic and/or non-genetic path is easier to follow: the baseline genetic and epigenetic heterogeneity; genetic stability; the efficacy and therapeutic window of the drug; the nature of the compensatory pathways that the drug induces; and the plasticity of the population. Cancers/tumours with higher genetic heterogeneity, lower epigenetic heterogeneity and/or low plasticity are more likely to use mutational change to adapt, whereas cancers/tumours with low genetic heterogeneity, high epigenetic heterogeneity and high plasticity, such as AML, are more likely to undergo non-genetic evolution.^29,30,36,74^ Different combinations of these variables will result in different relative contributions of genetic and non-genetic mechanisms in different cancer and treatment contexts.

The efficacy and therapeutic window of the drug can also influence the path to resistance, as genetic and non-genetic adaptation might cause different degrees of resistance. Therefore, a particular pathway would not be followed if it does not provide sufficient protection from the treatment. However, as the therapeutic window is usually limited, cancer cells do not always require a dramatic increase in drug resistance to be able to survive therapeutic pressure. In such instances, it is likely that the frequency of acquiring resistance, rather than the degree of resistance, will determine the adaptive pathway which is followed.^10^

This ‘path of most resistance’ model predicts that the resistance mechanism utilised by a given cancer to a given treatment should generally be predictable and reproducible. In line with this notion, modelling of resistance using the same patient-derived xenograft samples through multiple mice shows that some tumours invariably undergo non-genetic adaptation, while others always evolve by acquiring well-characterised resistance-conferring mutations.^21,30^ This supports the idea that in response to a particular therapy, specific tumours will generally follow the same path to acquired drug resistance.

## Conclusions and perspectives

The ability of cancer populations to use both genetic and epigenetic means to acquire therapeutic resistance exemplifies cancer’s remarkable resilience. Not only must our therapies overcome mutation-driven Darwinian evolution, but also non-genetic Darwinian adaptation and Lamarckian induction. At first, appreciation of this expanded adaptive arsenal might evoke depressing thoughts, but, if you consider that none of the current cancer therapies are designed or implemented in such a way as to counteract non-genetic adaptation, there is actually cause for hope. It is possible that many of our current approaches are inadequate at preventing non-genetic resistance and that the development of rational therapies/treatment strategies specifically designed to prevent these mechanisms of resistance, such as therapies that prevent compensatory pathways, might help to improve the poor survival outcomes of many cancers. Preliminary findings suggest that non-genetic adaptation is likely to be a pervasive and significant component of cancer resistance, therefore it is important that we carefully assess its relative contribution across a wide range of cancer and treatment contexts. Hopefully, such exploratory studies together with well-designed mechanistic experiments will help us decipher not only when, but also how and why, non-genetic resistance occurs. Only then do we have any chance of hitting this constantly moving target.

## Data Availability

Not applicable.
